# GSMN-TB: a web-based genome-scale network model of *Mycobacterium tuberculosis *metabolism

**DOI:** 10.1186/gb-2007-8-5-r89

**Published:** 2007-05-23

**Authors:** Dany JV Beste, Tracy Hooper, Graham Stewart, Bhushan Bonde, Claudio Avignone-Rossa, Michael E Bushell, Paul Wheeler, Steffen Klamt, Andrzej M Kierzek, Johnjoe McFadden

**Affiliations:** 1 School of Biomedical and Molecular Sciences, University of Surrey, Stag Hill, Guildford, Surrey, GU2 7XH, UK; 2 Tuberculosis Research Group, Veterinary Laboratories Agency (Weybridge), New Haw, Addlestone KT15 3NB, UK; 3 Max Planck Institute for Dynamics of Complex Technical Systems, Sandtorstrasse, D-39106 Magdeburg, Germany

## Abstract

**Background:**

An impediment to the rational development of novel drugs against tuberculosis (TB) is a general paucity of knowledge concerning the metabolism of *Mycobacterium tuberculosis*, particularly during infection. Constraint-based modeling provides a novel approach to investigating microbial metabolism but has not yet been applied to genome-scale modeling of *M. tuberculosis*.

**Results:**

GSMN-TB, a genome-scale metabolic model of *M. tuberculosis*, was constructed, consisting of 849 unique reactions and 739 metabolites, and involving 726 genes. The model was calibrated by growing *Mycobacterium bovis *bacille Calmette Guérin in continuous culture and steady-state growth parameters were measured. Flux balance analysis was used to calculate substrate consumption rates, which were shown to correspond closely to experimentally determined values. Predictions of gene essentiality were also made by flux balance analysis simulation and were compared with global mutagenesis data for *M. tuberculosis *grown *in vitro*. A prediction accuracy of 78% was achieved. Known drug targets were predicted to be essential by the model. The model demonstrated a potential role for the enzyme isocitrate lyase during the slow growth of mycobacteria, and this hypothesis was experimentally verified. An interactive web-based version of the model is available.

**Conclusion:**

The GSMN-TB model successfully simulated many of the growth properties of *M. tuberculosis*. The model provides a means to examine the metabolic flexibility of bacteria and predict the phenotype of mutants, and it highlights previously unexplored features of *M. tuberculosis *metabolism.

## Abstract 2

GSMN-TB, a genome-scale metabolic model of *M. tuberculosis*, was constructed and validated using experimental data.

## Background

Tuberculosis (TB), caused by *Mycobacterium tuberculosis*, is one of the most important diseases in the world today, being responsible for more than 8 million cases of disease each year and approximately 3 million deaths [1,2]. Control of human TB relies on vaccination, case finding, and chemotherapy. Current anti-TB drugs are relatively ineffective against 'persistent bacteria', and consequently prolonged treatment with combinations of drugs for 6 to 12 months is required to cure acute disease or eliminate persistent infections. The economic and logistic burden of administering TB treatment is enormous, particularly in industrially under-developed countries, where TB is most prevalent. A further complication in the treatment of TB is the emergence of multidrug-resistant strains of TB (both *M. tuberculosis *and *Mycobacterium bovis*) in many parts of the world [3,4]. Very few new classes of antibiotics have been approved for clinical use during the past decade. The exceptions (for instance, the oxazolidinones and daptomycin) are not applicable to TB infections. New anti-TB drugs are urgently required that shorten the duration of treatment, that have activity against drug-resistant strains, and that specifically target persistent cells.

An impediment to the rational development of novel drugs against TB is a general paucity of knowledge concerning the metabolism of *M. tuberculosis*, particularly during infection. One reason for this lack of knowledge is difficulty in applying biochemical techniques to the bacterium *in vivo*. In spite of this, several features of *in vivo *bacterial metabolism have been established. First, the essentiality of the glyoxylate shunt during intracellular growth indicates that *M. tuberculosis *survives by scavenging host lipids [5-7]. Second, there is growing evidence of a shift to anaerobic respiration during persistent infection [8-10]. These findings have been useful in directing rational drug development [11], but a more complete understanding of *M. tuberculosis *metabolism remains a major goal of TB drug research.

Availability of full genome sequences allows reconstruction of genome-scale metabolic reaction networks in micro-organisms. Metabolic capabilities of reconstructed networks consistent with stoichiometry of enzymatic conversions, their physiologic direction, and maximal allowable throughput can be studied by constraint-based computer simulation methods. These simulations provide a very useful framework in which to study metabolism in a systemic manner; they are also a novel approach to rational design of biochemical processes and drug discovery. Whole-genome metabolic network models of sequenced micro-organisms such as *Haemophilus influenzae *[12], *Escherichia coli *[13], *Helicobacter pylori *[14], and *Saccharomyces cerevisiae *[15] have proven to be useful in hypothesis generation and correction of errors in genome annotation, and have also been successful in predicting phenotypic behavior. These models, interrogated with various constraint-based computer simulation methods such as flux balance analysis (FBA) [16], elementary flux modes [17], or extreme pathways [18], provided information on the robustness of the metabolic networks and identified vulnerable pathways that may be targeted with novel drugs [19].

FBA has already been conducted in a network of reactions involved in mycolic acid synthesis [20] to identify TB drug targets. However, the network was limited to the fatty acid synthesis pathways and included just 28 enzymes. In this study we present the first reconstruction and constraint-based simulation of a genome-scale metabolic reaction network in *M. tuberculosis*. The model is calibrated by comparison with our experimental data on *M. bovis *bacille Calmette Guérin (BCG) growth in continuous culture. The model correctly predicted the growth phenotype of 78% of mutant strains in a published global mutagenesis dataset. Software allowing constraint-based simulations of *M. tuberculosis *metabolism via a web-based interface was developed in order to make our model available to the research community. This is the first reconstruction of a genome-scale metabolic reaction network published as a web resource, providing both data and interactive access to constraint-based simulation methods. We also demonstrate here that this model can be used to generate new hypotheses and thereby guide future research in the development of novel chemotherapeutics against TB.

## Results and discussion

### The genome-scale metabolic network of *M. tuberculosis*

The genome-scale metabolic network of *M. tuberculosis *(GSMN-TB) was constructed as described in the Materials and methods. The GSMN of *Streptomyces coelicolor *[21] was used as a starting point in the iterative model building process. *S. coelicolor *is an actinomycete that shares significant portions of genome synteny with *M. tuberculosis *[22]. The Kyoto Encyclopedia of Genes and Genomes (KEGG) gene orthology clusters were used to map the genes between two species and transfer corresponding metabolic reactions to the TB model. Of 849 unique reactions present in the final model, 487 (57%) were directly transferred from the *S. coelicolor *model following KEGG gene orthology mapping. This preliminary model has been further supplemented by data from KEGG and BioCyc databases.

A significant proportion of the model could not be constructed using semi-automatic methods and was therefore generated by analysis of original research articles. Table 1 lists these unique *M. tuberculosis *metabolic pathways, including those relevant to the synthesis of the cell envelope of *M. tuberculosis*, which contains a diverse array of complex lipids and carbohydrates that are important for growth and pathogenesis, and are important drug targets. Because fatty acid metabolism is thought to be a crucial factor in TB pathogenesis [23], standard biochemical pathways for β-oxidation of fatty acids pathways were added, including additional reactions for catabolism of odd and even numbered fatty acids and unsaturated fatty acids. Respiratory pathways and synthesis of biomolecules specific to mycobacteria were also modeled by manual annotation. Transport reactions included those responsible for the the import of minerals, carbon, nitrogen and high molecular weight compounds such as biotin. Transport reactions for long chain fatty acids such as palmitate and oleic acid were also included because there is evidence that *M. tuberculosis *consumes host-derived lipids *in vivo *[23]. Iron metabolism is also an important component of the pathogenesis of many microbes, including *M. tuberculosis *[24]. We simulated a requirement for iron by allowing ferric ion transport (both citrate and mycobactin mediated) and incorporating iron into the heme group of cytochromes such that it cycles between the ferric and ferrous valence states according to the oxidation state of the electron carrier.

**Table 1:** Metabolic pathways that have been modelled by direct annotation of original literature data

Pathway	References
Biosynthetic pathways	
Arabinogalactan	[60,61]
Mycolic acids	[62]
Trehalose monomycolate, trehalose dimycolate	[63]
Dimycocerosate esters (DIMs)	[64-66]
Phenolic glycolipid (PGL)	[67]
Sulfolipid SL-1	[68-70]
Phosphatidylinositol mannosides (PIMS)	[71]
Lipomannan (LM)	
Lipoarabinomannan (LAM)	
Mannosyl β-1-phosphodolichol (MPD)	[72]
Siderophore mycobactin	[73]
Co-factor F420	[74]
Mycothiol	[75]
Catabolic pathways	
Additional beta oxidation pathways	
Odd and even numbered fatty acid catabolism	
Respiratory pathways	
NADH dehydrogenases, cytochromes	[76,77]
Nitrate as an alternative electron acceptor	[78]

*M. tuberculosis *is a facultative intracellular parasite that is capable of growth within host cells, in the extracellular milieu, and *in vitro*. Biomass composition data are available only for *in vitro *grown *M. bovis *BCG, and so this was used to model the *M. tuberculosis *cell for the *in silico *model. However, it is well established that many of the outer cell wall components of *M. tuberculosis *(such as phenolic glycolipid), although produced *in vitro*, are not essential for *in vitro *growth but are required for pathogenesis. In order to make the model applicable to *M. tuberculosis *grown both *in vitro *and *in vivo*, we therefore defined two biomass components based on published experimentally derived values for macromolecular composition of *M. tuberculosis*. (See Additional data files 1 to 3: Additional data file 1 illustrates the estimated macromolecular composition for *M. tuberculosis*, Additional data file 2 shows the calculations used to estimate that composition, and Additional data file 3 shows the conversion between stoichiometric formulae and mmol/l per gram of biomass.) The first (BIOMASS1) reflects the actual macromolecular composition of *M. tuberculosis*. The second (BIOMASSe) is a minimal macromolecular composition of *M. tuberculosis *and includes only those components (DNA, RNA, protein, essential co-factors, and cell wall skeleton) that are thought to be essential for *in vitro *growth. It is this second biomass that was used to make predictions regarding gene essentiality *in vitro*. To simulate the requirement of co-factors for nonessential reactions, we introduce the concept of a 'replenishing flux', in which the co-factors are included in reactions but with a low (0.001), unbalanced stoichiometric coefficient toward consumption, forcing co-factor synthesis only when the co-factor utilizing reaction is active.

The final model contains 849 reactions and 739 metabolites, and involves 726 genes (Table 2). These numbers refer to unique stoichiometric formulae, because paralogous genes, involved in the same reaction, were accounted for by Boolean statements describing gene-protein associations, rather than being modeled by duplication of reactions (see Materials and methods). The reaction formulae, FBA parameters, and gene-protein associations are summarized in Additional data files 4 (reaction formulae, limits, Enzyme Commission (EC) numbers, genes, and pathway classifications), 5 (references for those reactions), and 6 (metabolite names).

**Table 2:** Statistics of the GSMN-TB model

Reaction Class	Number
Enzymatic conversions	723
Transport reactions	126
Total number of reactions	849
Orphan reactions	210
Genes	726
Internal metabolites	638
External metabolites	101
Total number of metabolites	739

GSMN-TB, genome-scale metabolic network of *M. tuberculosis*.

### Quantitative calibration and validation of the GSMN-TB model

#### Quantitative calibration of the model

The quantitative results of FBA of the GSMN-TB model depend on the three global energetic parameters, which are not explicitly accounted for by currency metabolite production/consumption included in the stoichiometry of individual enzymatic reactions. Specifically, these parameters are as follows: the ratio of the number of ATP molecules formed to the number of O atoms reduced (P/O ratio); the cost of polymerization of the building blocks into biologic polymers (DNA replication, transcription, translation, and so on); and ATP costs for growth-associated maintenance (see Materials and methods, below). These parameters must either be measured or calibrated by comparison of the model predictions with experimental data. For well established model systems such as *Escherichia coli *there is a plethora of metabolic flux data available from steady-state chemostat cultivations, which allows reliable estimation of energetic parameters. The slow growth rate of pathogenic mycobacteria, combined with problems associated with clumping of this group of bacteria and safety considerations, has created obstacles for researchers attempting chemostat cultures of these strains. As a result, quantitative metabolic flux data for *M. tuberculosis *group organisms are limited to the findings of chemostat experiments included in our previous report [25] and the Additional data files presented here.

**Figure 1:**
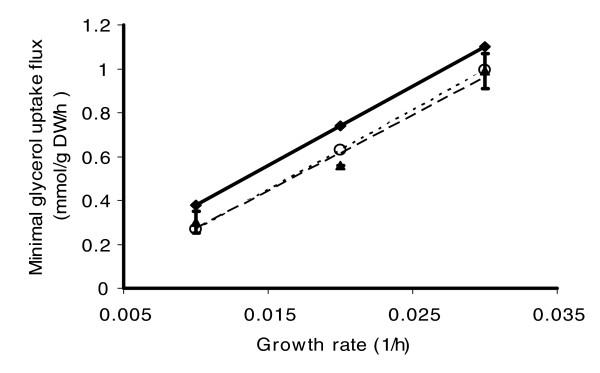
Comparison of predicted and measured glycerol uptake rates as a function of controlled growth rate. Triangles indicate experimentally measured glycerol uptake rates for three growth rates set by three different dilution rates in the chemostat model. The dashed line represents the linear function fitted to the experimental data. Diamonds and solid line represent predictions of the model if glycerol were the only carbon source. Circles and dotted line show predictions of the model when additional oleic acid (hydrolysis product of Tween 80) transport in the range of 0 to 0.04 mmol/g dry weight (DW) per hour was allowed.

Experimental data obtained for growth of *M. bovis *BCG in glycerol-limited continuous culture at three growth rates were compared with the quantitative predictions of the GSMN. BCG and *M. tuberculosis *have a high degree of homology, sharing 99.9% of DNA, and possess identical metabolic pathways for utilization of glycerol [26]. FBA minimization of glycerol consumption at fixed growth rates was simulated by setting the P/O ratio to 1 and the ATP dissipation flux due to polymerization of biomolecules to 1.0 mmol/g dry weight (DW) per hour, and consumption of 47 mmol/g DW ATP for maintenance was added to the biomass formation reaction. These values were set using data obtained from related bacteria [21,27], because no data were available from mycobacteria. However, it is demonstrated below that gene essentiality predictions and other important qualitative insights into TB biology generated by this model are not affected if the energetic parameters are varied within the range of values reported for different microbial species. The resulting plot (Figure 1) demonstrates that the predicted biomass production yield (reciprocal of the slope of the line) was within the 95% confidence interval of the experimental value. However, predicted glycerol consumption rates were higher than the experimentally determined values. This discrepancy could not be resolved by testing different values of the three energetic parameters in the ranges reported for different microbial species (data not shown).

A possible explanation of the discrepancy between the predicted and experimental data is that BCG cells consumed carbon from an additional source. Although glycerol is the main carbon source in Roisin's minimal medium, Tween 80 is also present in the culture medium to reduce cell clumping. Tween 80 is an oleate ester of sorbitol, with an oleate content above 75%, and minor amounts of other unsaturated and saturated fatty acids. The tubercle bacillus is known to be able to hydrolyze Tween 80 and can also utilize the fatty acids released as a sole carbon source [26]. The FBA simulation was repeated with minimization of glycerol uptake flux and oleic acid transport flux constrained in the range of 0 to 0.04 mmol/g DW per hour. The resulting plot (Figure 1) demonstrates that the predicted line is contained within 95% confidence (both slope and intercept) intervals of experimentally measured values at experimentally reasonable oleic acid consumption rates. Preliminary nuclear magnetic resonance analysis (data not shown) on spent culture media are also consistent with the hypothesis that Tween 80 was being assimilated under the conditions of the experiment and contributing to the biomass yield.

#### Validation of the model by comparison with global mutagenesis data

To evaluate the predictive power of the model we compared *in silico *predictions of gene essentiality with the findings of a previously reported global mutagenesis study of gene essentiality in *M. tuberculosis *by transposon site hybridization (TraSH) [28]. The TraSH technique combines high-density transposon mutagenesis with microarray mapping of pools of mutants, which allows rapid determination of the full repertoire of genes required for growth under given environmental conditions.

It is well established that many of the macromolecular components of *M. tuberculosis*, although essential for virulence, are not required for *in vitro *growth. For *in vitro *gene essentiality predictions, we therefore used BIOMASSe as the objective function of the GSMN-TB; BIOMASSe is a minimal biomass composition that reflects current knowledge of the biomass components of *M. tuberculosis *that are essential for growth *in vitro*. To model the composition of the minimal media Middlebrook 7H10 used in the TraSH experiment of Sassetti and coworkers [28] we simulated the transport or secretion of the following external metabolites in the model: glucose, glycerol, iron (citrate-mediated iron transport), ammonia, nitric dioxide, phosphate, sulfate, oxygen, carbon dioxide, molybdenum, and biotin.

Theoretical predictions were generated by removing single genes from the GSMN-TB (*in silico *mutation) and calculating the resulting maximum growth rate for each *in silico *mutant. We emphasize, however, that this predicted maximum growth rate should be viewed solely as a qualitative prediction. Our aim was to identify genes that prevented or severely compromised the capacity to synthesize biomass, which would lead to zero or greatly reduced growth rates in the GSMN-TB. Most mutations had little or no effect on growth rate, but some *in silico *mutations were lethal (in the sense that the resulting maximum growth rate was zero) or depressed growth rate to values between zero and the maximum predicted growth rate for the 'wild type'. To identify essential genes we set an arbitrary growth rate threshold (see Materials and methods, below) such that mutants with a maximum predicted growth rate below that threshold were considered to be essential for growth. (Below, we examine the effect of varying the growth rate threshold on prediction accuracy.)

The lists of essential and nonessential genes predicted by the model were compared with essentiality assignment according to the previously reported TraSH analysis [28]. Note that in the TraSH study gene essentiality predictions were based on the ratio of the microarray hybridization signal obtained from labeled insertion sites in a saturated transposon mutant library compared with a control of labeled genomic DNA. This ratio reflects the relative abundance of each transposon mutant in the TraSH library. Genes with microarray signal ratios of less than 0.2 were predicted to be essential. We designate this cut-off value as the TraSH threshold. GSMN-TB and TraSH-based gene essentiality assignments were compared and the numbers of true-positive (essential both in the model and experiment), false-positive (essential in the model, nonessential in experiment), true-negative (nonessential in the model and experiment), and false-negative (nonessential in the model, essential in experiment) predictions were computed (Table 3).

**Table 3:** Comparison of theoretical gene essentiality predictions with results of TraSH experiment *in vitro*

	TraSH threshold of 0.2	TraSH threshold of 0.1	TraSH threshold of 0.1, altered energetic parameters
True positive	154	115	114
False positive	71	110	110
True negative	385	432	432
False negative	95	48	49
Sensitivity	62%	71%	70%
Specificity	84%	80%	80%
Correct predictions	76%	78%	77%
*P*^a^	< 2.2 × 10^-16^	< 2.2 × 10^-16^	< 2.2 × 10^-16^

^a^Fisher exact test. TraSH, transposon site hybridization.

**Figure 2:**
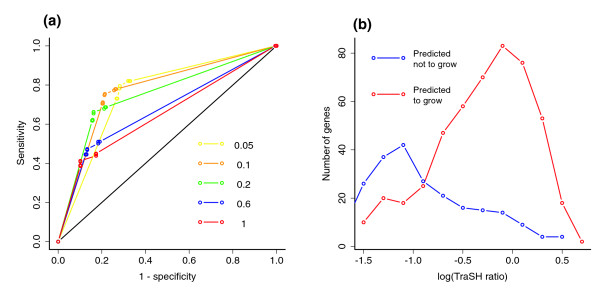
Comparison of gene essentiality predictions with TraSH data for *in vitro *growth on Middlebrook 7H10 medium. **(a) **Dependence of prediction results on the model and experimental thresholds for declaring gene essentiality. The plot shows receiver operating characteristic (ROC) curves for different transposon site hybridization (TraSH) ratio thresholds for determination of essential genes in experimental data. Each ROC curve shows 100 points corresponding to sensitivity and specificity of the model predictions obtained for growth rate thresholds varying in the range from 0.0 to 0.1 (increment 0.001). The growth rate threshold has little effect on prediction parameters. For values greater than 0.052 all genes were declared essential. Any threshold in the range from 0.001 to 0.041 resulted in exactly the same gene essentiality predictions. The ROC curve closest to the best theoretically possible prediction (sensitivity and specificity equal to 1) was obtained for a TraSH ratio threshold of 0.1. **(b) **Distributions of the hybridization ratio of the TraSH library to genomic DNA signal recorded in TraSH experiment for genes present in the model. Blue line shows distribution of the TraSH ratio among the genes that were predicted by the model to be essential for growth. Red line shows distribution of TraSH ratio among genes predicted to be nonessential for growth. Medians of the two distributions are significantly different by means of the Mann-Whitney test (*P *< 2 × 10^-16^). Thus, the genes that are predicted to be essential have significantly lower median value of insertion probe to genomic probe ratio than genes predicted to be nonessential. This is in accordance with experimental data, because the low ratio indicates that inactivation of the target gene by transposon insert results in depletion of the mutant strain after the growth on Middlebrook 7H10 medium.

In order to visualize the influence of the two thresholds (growth rate threshold and TraSH threshold) on the sensitivity and specificity of the GSMN-TB predictions, receiver operating characteristic (ROC) curves were plotted (Figure 2a). The ROC curves (Figure 2a) demonstrated that varying the growth rate threshold had little effect on either sensitivity or selectivity. This is a consequence of the fact that most *in silico *mutants had either a predicted growth rate that was the same as the wild type or a predicted growth rate of zero. In contrast, varying the TraSH threshold had a marked effect on the prediction parameters (Figure 2a). The ROC curve corresponding to the TraSH threshold of 0.1 was closest to the best possible prediction result (sensitivity and selectivity of 1). The curve obtained for the TraSH threshold of 0.2 (the value used in the reported study [28]) exhibited lower sensitivity and a slightly lower number of correct predictions. The results of the comparison of essentiality predictions for individual genes with the previously published *in vitro *TraSH data [28], using a growth rate threshold of 0.001 and TraSH ratio thresholds of either 0.1 or 0.2, are shown in Table 3.

The GSMN-TB model predicts that approximately 34% of *M. tuberculosis *genes in the model are essential for growth in minimal Middlebrook 7H10 media, which is very close to the estimated value of 35% essential genes in *M. tuberculosis *[29]. The number of true predictions was significantly higher than expected by chance (Fisher exact test; *P *< 2.2 10^-16^). The overall fraction of correct predictions is 78%, with sensitivity and specificity of 71% and 80%, respectively, if a TraSH ratio threshold of 0.1 is applied. Predictions are robust with respect to the quantitative parameters of the FBA model. When energetic parameters were set to 1 (P/O ratio), 5.0 mmol/g DW per hour (ATP dissipation), and 60 mmol/g DW per hour ATP molecules (growth-associated maintenance), the result changed for only one gene (a true positive becomes a false negative). Therefore, the prediction accuracy was not affected by substantial change in energetic parameters.

To validate further the predictive power of the model, the distributions of TraSH hybridization signal (TraSH probe/genomic probe) were plotted for both essential and nonessential genes as predicted by the model (growth rate threshold of 0.001; Figure 2b). Medians of the two distributions are significantly different (Mann-Whitney test; *P *< 2.2 10^-16^). The genes predicted to be essential have significantly lower TraSH hybridization ratios than genes predicted to be nonessential. This is in accordance with the experimental data. This demonstrates the predictive power of the model using an approach that is independent of the TraSH signal ratio threshold.

#### Validation of the model by comparison with literature data on phenotypes of single gene knockouts

Some of the discrepancies identified between the FBA predictions and the global mutagenesis data can be attributed to an undefined level of inaccuracy in TraSH assays because there are several examples in which the *in silico *predictions are validated by individual gene knockout studies. The *inhA *gene, which is the known drug target for the key antituberculous drug isoniazid [30] and has been shown to be essential in the related *Mycobacterium smegmatis *[31], was nonessential in the TraSH experiment (TraSH ratio 0.38) but was correctly predicted to be essential for growth by the GSMN-TB model. Many false-negative genes (nonessential in the model but essential in global mutagenesis data) may be due to gene regulation of isoenzymes. Both menaquinol oxidase systems (the aa3-type and bd-type) are predicted to be nonessential because they are functionally redundant in the model. However, the apparent essentiality (false-negative prediction) of genes encoding the aa3-type cytochrome c oxidase indicates that this system is likely to be the main electron transport system operating in the aerobic conditions in which the global mutagenesis experiment was performed.

As a further check of the accuracy of the GSMN-TB, we compared (Table 4) the phenotype of known individual gene knockout mutants (sometimes in related organisms, such as *M. smegmatis*) with gene essentiality prediction by both TraSH result and GSMN-TB. (All genes whose inactivation reduced growth rate were designated GSMN-TB essential; this was recorded as a correct prediction if the gene knockout mutant exhibited temperature sensitivity, slow growth, or auxotrophy.) As can be seen in Table 4, out of 29 genes examined the GSMN generated a correct prediction for 20 genes, whereas TraSH generated the correct prediction for 22 genes. GSMN-TB and TraSH yielded discordant predictions for eight genes: GSMN-TB gave the correct prediction for three of those genes and TraSH generated the correct prediction for five genes. Errors in GSMN-TB predictions were immediately informative in suggesting model revisions. For instance, *mshB *and *mshC *are both involved in mycothiol synthesis, which is nonessential in the GSMN-TB because mycothiol is currently not a biomass component and neither is it required for the synthesis of any biomass component. The essentiality of *mshC *and poor growth of *mshB *indicate that mycothiol should be included as either a biomass component or an essential co-factor for synthesis of a biomass component, or both.

**Table 4:** Comparison of TraSH and GSMN-TB predictions of gene essentiality with experimentally determined phenotype

Gene	Prediction		Knock-out mutant		Reference
		
	GSMN-TB	TraSH	Species	Phenotype	
*AftA*	E	E	*C. glutamicum*	Slow growth	[60]
*AroK*	E	E	*M. tuberculosis*	Essential	[79]
*Ask*	E	E	*M. smegmatis*	Auxotroph	[80]
*CysH*	E	E	*M. smegmatis*	Auxotroph	[81]
*GlnA1*	E	E	*M. tuberculosis*	Essential	[82]
*GlnA3*	NE	NE	*M. tuberculosis*	Nonessential	[82]
*GlnA4*	NE	NE	*M. tuberculosis*	Nonessential	[82]
*hemZ*	E	E	*M. tuberculosis*	Essential	[83]
*InhA*	E	NE	*M. smegmatis*	Ts lethal	[31]
*ino1*	NE	NE	*M. tuberculosis*	Auxotroph	[84]
*KasA*	NE	E	*M. smegmatis*	Essential	[85]
*LeuD*	E	E	*M. tuberculosis*	Auxotroph	[86]
*LysA*	E	E	*M. tuberculosis*	Auxotroph	[87]
*manA*	E	E	*M. smegmatis*	Hyperseptation and loss of viability	[88]
*mshB*	NE	NE	*M. tuberculosis*	Grows poorly	[89]
*mshC*	NE	E	*M. tuberculosis*	Essential	[90]
*murD*	E	E	*M. tuberculosis*	Nonessential	[82]
*murI*	E	NE	*M. tuberculosis*	Nonessential	[82]
*Ndh*	NE	NE	*M. smegmatis*	Ts lethal	[91]
*NrdF2*	E	E	*M. tuberculosis*	Essential	[92]
*OtsA*	E	E	*M. tuberculosis*	Slow growth	[93]
*OtsB2*	NE	E	*M. tuberculosis*	Essential	[93]
*panCD*	E	NE	*M. tuberculosis*	Auxotroph	[94]
*panCD*	E	E	*M. tuberculosis*	Auxotroph	[94]
*PimA*	E	NE	*M. smegmatis*	Essential	[95]
*PurC*	E	E	*M. tuberculosis*	Auxotroph	[96]
*Purl*	E	E	*M. tuberculosis*	Auxotroph	[96]
*RmlB*	NE	E	*M. smegmatis*	Essential	[97]
*TreS*	NE	NE	*M. tuberculosis*	Nonessential	[93]

Shown is a comparison of transposon site hybridization (TraSH) and genome-scale metabolic network of *M. tuberculosis *(GSMN-TB) predictions of gene essentiality with experimentally determined phenotype for genes that have been investigated by specific gene knockout. E, essential; NE, nonessential.

#### Prediction of gene essentiality for known drug targets

The GSMN-TB contains five genes that encode enzymes that are drug targets: *inhA *(isoniazid and ethionamide), *fasI *(pyrazinamide), *embAB *(ethambutol), *ddlA *(cycloserine), and *alr *(cycloserine). All of these genes were correctly predicted as essential for growth on 7H10. This demonstrates the utility of the GSMN-TB in identifying potential drug targets in metabolic reactions

### Use of the GSMN-TB to explore the metabolic state of *M. tuberculosis*

An important application of the GSMN-TB is to model the metabolic state of *M. tuberculosis*, particularly in situations that are difficult to approach experimentally, such as during infection. *M. tuberculosis *is a versatile chemoheterotroph that can utilize a wide range of sources of carbon and nitrogen. Similarly, the *in silico *model is able to generate feasible solutions to optimize biomass or 'grow' on a range of carbon and nitrogen sources. Feasible flux distributions include expected biochemical pathways; for instance, most of the flux from glucose is directed through glycolysis and the tricarboxylic acid (TCA) cycle, whereas the glyoxylate shunt is utilized for growth on acetate (or fatty acids). The GSMN-TB also indicated that *M. tuberculosis *has much more metabolic flexibility than is generally accepted. For example, TraSH data [28] demonstrated that several enzymes of the TCA cycle, including malate dehydrogenase, were nonessential, and this was also predicted by the model. When malate dehydrogenase was inactivated *in silico *using the GSMN-TB, the resulting carbon flux was predicted to be shunted through the anaplerotic reactions catalyzed by malic enzyme, pyruvate phosphate dikinase, and phosphoenolpyruvate carboxykinase.

**Figure 3:**
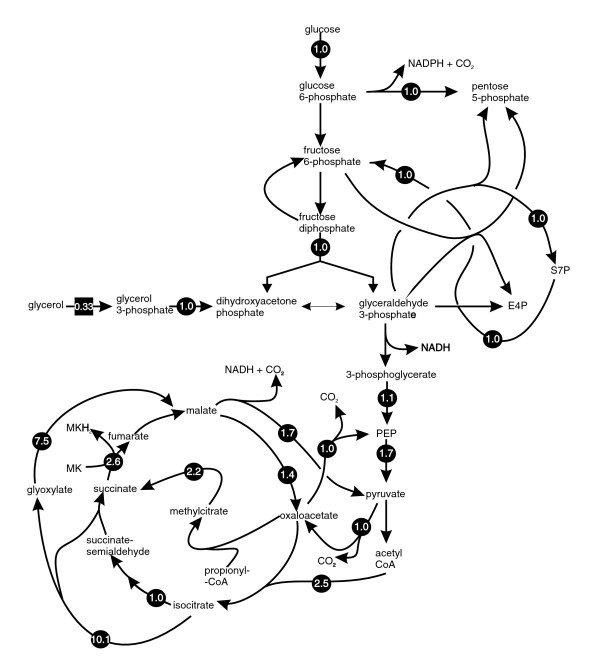
Predicted response of the *Mycobacerium tuberculosis *to slower growth rate induced by carbon limitation. Only selected central metabolic pathways are illustrated. The slower growth rate was simulated by adjusting glycerol uptake rates to obtain a predicted growth rate of 0.03 (fast growth rate corresponding to doubling time of 23 hours) and 0.01 (slow growth rate corresponding to doubling time of 69 hours). Arrows indicate biochemical reactions or pathways, and the number on the arrow indicates the response of the genome-scale metabolic network of *M. tuberculosis *(GSMN-TB) to slower growth rate. The numbers were calculated by flux variability analysis (FVA) as the ratios of midpoints of flux ranges obtained for slow and fast growth rates. The values have been normalized to account for the lower absolute carbon flux values at the slower growth rate, except for the glycerol uptake rate, which is not normalized to emphasize the fact that the growth rate was reduced by limiting glycerol. The direction of the arrows indicates the direct of flux, not reaction reversibility. CoA, coenzyme A; E4P, D-erythrose-4-phosphate; MK, menaquinone; MKH, menaquinol; S7P, D-sedoheptulose-7-phosphate.

In order to investigate the value of the model as a hypothesis generating tool, we analyzed the *in silico *metabolic response of *M. tuberculosis *to slow growth, because this is a key component of persistence/dormancy in *M. tuberculosis*. We compared the predicted flux ratios for two different growth rates that could be experimentally verified in a chemostat. A doubling time of 23 hours (dilution rate 0.03) was compared with a doubling time of 69 hours (dilution rate 0.01). Flux ratios for central metabolism (0.01/0.03) were calculated by flux variability analysis (FVA) as the ratios of midpoints of flux ranges obtained for slow and fast growth rates (Figure 3). Although it should be emphasized that these predictions are qualitative in nature, the majority of the flux values were close to unity, indicating that the relative fluxes are unchanged. However, some reactions have markedly different flux predictions in the two growth rates, including reactions that are involved in the glyoxylate shunt. There was a large predicted increase in flux through the isocitrate lyase reaction. This prediction suggested the hypothesis that isocitrate lyase was involved in maintaining growth at slow growth rates. To investigate this hypothesis we measured the activity of isocitrate lyase activity in BCG cells grown at both growth rates in a chemostat. In accordance with predictions, specific isocitrate lyase activity was significantly higher (twofold change; *t*-test, *P *= 0.0002) in the slow growing cells (Table 5).

**Table 5:** *In vitro *isocitrate lyase activities in crude extracts of chemostat cultivated BCG cells

Dilution rate (per hour)	Specific activity
0.01	42.67 ± 2.68
0.03	21.20 ± 0.69

Results represent the average values ± standard deviations from three independent measurements. The unit of enzyme activity is nmol/min per mg protein.

### The online resource for analysis of *M. tuberculosis *metabolism

We have created web-based software that allows online access to data files and computational methods used for constraint-based simulations of the GSMN-TB model of TB metabolism. This is the first GSMN model published as an interactive resource allowing the scientific community to interrogate the model with biologic data. The web server presents the most recent version of the model and will be continuously updated as more metabolic genes are identified and characterized. The current version of the system implements the following computational methods. The FBA method computes the maximal theoretical growth rate under given experimental conditions and one of the possible metabolic flux distributions sustaining maximal growth rate. To allow further exploration of the metabolic state of the cell, we have also implemented FVA. The FVA method determines the minimal and maximal flux for each reaction in the system that is consistent with this maximal theoretical growth rate (see Materials and methods, below). In contrast to the flux distribution computed in a single FBA simulation, the FVA flux ranges are unique. Our server also allows gene and reaction essentiality predictions.

All calculations described above can be performed for a variety of experimental conditions. The user is able to specify media conditions by changing the bounds of the GSMN transport reactions (most of the transport reactions in the GSMN-TB are currently constrained to zero). Both model file and results are displayed in tabular format, with the gene annotation linked to the TubercuList database [32].

We have also implemented methods to investigate the *in vivo *growth of *M. tuberculosis *using the web-based software. The use of FBA to model *in vivo *growth is more problematic because it is not clear what to use as an objective function for optimization. We have tackled this problem by including two objective functions that can be optimized: one utilizes a minimal biomass composition, which includes only those components that are thought to be essential for *in vitro *growth; and the other uses a 'complete' biomass composition, which includes synthesis of macromolecular components (virulence factors), such as dimycocerosate esters and sulfolipid, that are thought to be essential for infection. This allows the user to model both *in vitro *and *in vivo *growth and, for instance, to predict genes that are only essential for growth *in vivo*.

Constraint-based computer simulations methods available in our software are computationally fast enough to allow interactive online work. Results of FBA and single gene essentiality predictions appear instantaneously in the user's browser, and FVA results are computed in less than 10 min.

**Figure 4:**
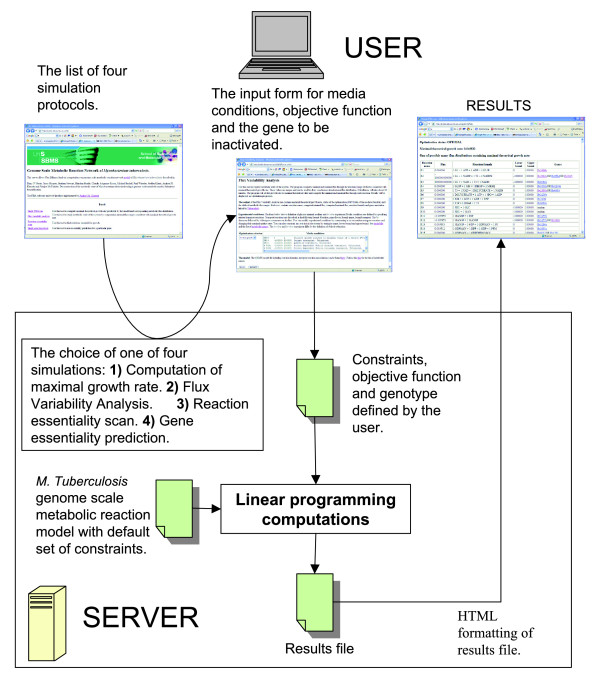
Overview of interactive resource workflow of GSMN-TB. The user chooses one of four analysis protocols (computation of maximal growth rate, flux variability analysis [FVA], reaction essentiality scan, or single gene essentiality prediction). Depending on the choice, an appropriate input form is presented. The server runs linear programming using the genome scale metabolic reaction network of *Mycobacterium tuberculosis*. Constraints defined by the user overwrite the default set of constraints specified in the model file. Numerical results are formatted as HTML and sent to the user's browser. GSMN-TB, genome-scale metabolic network of *M. tuberculosis*.

**Figure 5:**
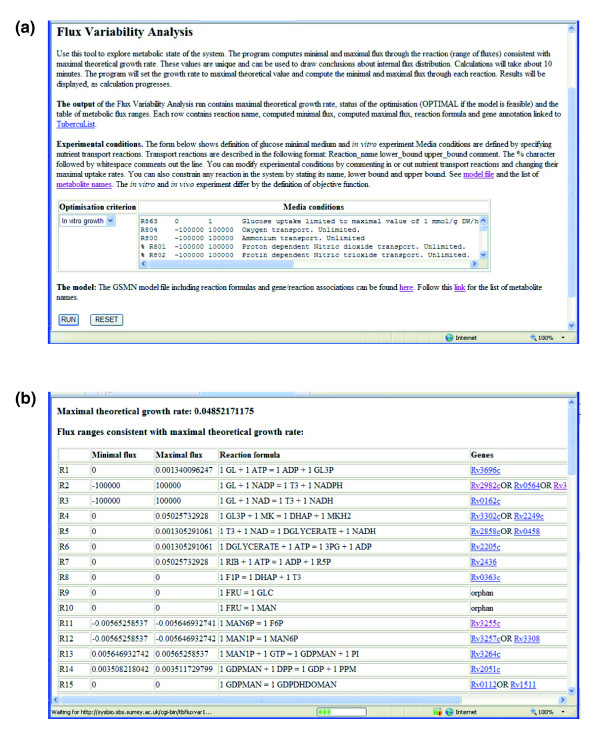
Screenshots illustrating FVA of *M. tuberculosis *using GSMN-TB. **(a) **Simulation set up. User specifies media conditions by setting minimal and maximal capacities of transport reactions in the 'Media conditions' field. The field contains specification of minimal glucose media and lists transport reactions that can be included by removing comment character. The page contains the link to the full model file, and the user may identify other reactions to be constrained. This adds additional flexibility to the simulation set up. The user may also choose one of the two objective functions used in our model to simulate *in vitro *or *in vivo *growth requirements. **(b) **Result of flux variability analysis (FVA) simulation. Maximal theoretical growth rate is displayed at the top of the page. Each row in the table contains reaction name maximal and minimal flux consistent with the maximal theoretical growth rate, reaction formula, and gene annotation. Gene names are linked to genome annotation pages of the TubercuList database. The rows of the table are loaded as computation progresses. The time of the simulation is about 10 min. GSMN-TB, genome-scale metabolic network of *M. tuberculosis*.

The web interface to our interactive resource is now available [33]. Figures 4 and 5 show the workflow of the software and screenshots from the interface. More detailed presentation of the interface can be found in the manual (Additional data file 7).

## Conclusion

We have built the first genome-scale metabolic network (GSMN) model of the tubercle bacillus, which is the agent responsible for approximately 5% of all deaths worldwide and 9.6% of all adult deaths. The model incorporates nearly all known biochemical reactions of the micro-organism and describes the biosynthetic pathways that lead to the synthesis of all of the major macromolecular components, including known virulence factors. The model provides new insights into the biology of the pathogen and provides a framework for integrating metabolic, proteomic, and transcriptomic data. Thereby, it can serve as a platform on which to build extended models of the *M. tuberculosis *cell, including all levels of biochemical network organization.

To be representative, systems level models must be constrained with experimental data. The model was therefore calibrated using our data from chemostat cultivations of *M. bovis *BCG. FBA simulations predicted a consistently higher rate of glycerol consumption than was observed. The most likely explanation for this is that the cells are simultaneously utilizing both glycerol and oleic acid (derived from hydrolysis of Tween 80) as a carbon source. This pattern of mixed substrate utilization is in contrast to the more extensively studied diauxic growth that is typical of batch-grown micro-organisms, in which the substrate supporting the greatest growth rate is utilized first and the second substrate is only consumed after exhaustion of the preferred substrate. However, mixed substrate utilization has been shown to operate in carbon-limited chemostat cultures of organisms such as *E. coli *that demonstrate diauxic growth in batch culture [34]. It is likely that the pattern of low availability of mixed substrates is closer to situations that pertain in most natural environments than the high single substrate conditions that are most often studied in batch culture [35].

The accuracy of the GSMN-TB model of *M. tuberculosis *was tested by comparison of model predictions of gene essentiality with global mutagenesis (TraSH) experimental data. The model was shown to have a high degree of accuracy, correctly predicting the phenotype for more than 75% of single gene mutants. Discrepancies between the model and TraSH mutagenesis data were also informative. In some cases the model prediction matched the phenotype of individual gene knockout studies more closely than the TraSH mutagenesis data. This was true for the *inh*A gene, whose product is a target for the key antituberculous isoniazid. This result verifies the use of the model as a tool for drug discovery. In addition to known drug targets, the model predicts 220 essential genes in *M. tuberculosis*, any one of which is a potential target for new antituberculous drugs. The remaining 24% discordant predictions (174 genes) clearly must be investigated further in a reiterative cycle of hypothesis generation, experiment, model improvement, and further experimentation. Identification of discrepancies between model predictions and experimental data are informative in that they indicate errors in the model, errors in gene annotation, or incomplete knowledge of *M. tuberculosis *metabolism, such as the presence of an unknown isoenzyme.

The model is also an excellent tool for mining existing datasets, for instance those resulting from TraSH mutagenesis studies examining gene essentiality in different environments. Interrogation and integration of datasets such as global mutagenesis data can thereby be used to refine further the model in an iterative process. The genome-scale model has considerable advantages over traditional genome annotation and pathway databases, including its internal stoichiometric consistency, systems level integration, and its ability to predict gene essentiality for different media conditions automatically. The model inputs data such as growth characteristics of particular genotypes to auto-generate hypotheses in the form of predicted flux maps of internal metabolism. In addition, the model provides a platform that could be used to integrate and manage 'omics data in a manner that is consistent with the underlying biochemistry and genetics of the organism [14,15,36,37]. Moreover, the lists of genes and reactions predicted by the model to be essential for growth, under given media conditions, may easily be combined with other drug target prioritization protocols, which account for the availability of structural information about the enzyme, availability of its inhibitors, and sequence similarity to host and other bacterial proteins [38].

The constraint-based simulation methodology, used in this work and implemented in the GSMN-TB server, is currently the most practical solution for studying metabolic flux distribution in the genome-scale metabolic reaction networks. This method involves optimization of the objective function represented by one of the fluxes in the network, usually the flux to biomass that determines growth rate. Although it could be argued that optimization of growth rate is not appropriate to the very slow growing *M. tuberculosis*, our results indicate that - under the carbon-limited conditions we tested - the organism uses carbon source efficiently with yields close to the maximal theoretical values predicted by the model. However, the conclusions presented in our work are qualitative and do not depend on whether the objective function flux actually reaches its maximal/minimal possible value. We demonstrated that gene essentiality predictions were not sensitive to large changes in the growth rate thresholds used for viability predictions in mutants. Similarly, the findings of the FVA of *M. tuberculosis *growing at two different growth rates should be considered qualitative rather than quantitative predictions.

In addition to validating the model, we also demonstrated its potential to generate experimentally testable hypothesis by predicting the metabolic response of *M. tuberculosis *to carbon-limited slow growth. Persistence is a central feature of the biology of *M. tuberculosis*, being responsible both for latency and the necessity for long treatment regimens [39]. Little is known about the physiologic state of *M. tuberculosis *during persistence, but slow or zero growth is generally thought to be a key property. *In vitro *models of persistence invariably involve slowing the growth rate through, for instance, oxygen limitation [40] or nutrient starvation [41]. We therefore investigated the *in silico *response of the tubercle bacillus to slow growth in our model by FVA. The most significant alteration was a predicted increased flux through the glyoxylate shunt, particularly the isocitrate lyase reaction, at slow growth rate. This hypothesis was supported experimentally by the demonstration that isocitrate lyase activity was higher in slow (doubling time 69 hours) growing BCG cells than in faster (doubling time 23 hours) growing cells. This finding is consistent with the model flux prediction and thereby supports the hypothesis that isocitrate lyase plays a specific role in slow growing mycobacteria. Several pathogens, including *M. tuberculosis*, require isocitrate lyase for long-term persistence in the host [42-45]. Increased isocitrate lyase activity has previously been reported in the Wayne (oxygen-limited) *in vitro *model of *M. tuberculosis *persistence [46]. In addition, Muñoz-Elías and McKinney [6] demonstrated that inactivation of the isocitrate lyase genes of *M. tuberculosis *led to attenuation for survival and multiplication in mice and macrophages, but this finding has generally been interpreted as indicating a role for fat catabolism in survival of this pathogen in the host. Our results indicate that isocitrate lyase may play a more general role in the slow growth of *M. tuberculosis*, irrespective of the means of growth limitation. This finding could have implications for drug development, because isocitrate lyase has been intensively investigated as a potential antitububerculous drug target [7,11,47].

The application of constraint-based modeling to the *in vivo *situation is of course more challenging than simulation of *in vitro *growth. Because the pathogen probably does not maximize growth rate during infection, an objective function based exclusively of maximization of growth rate is unlikely to be entirely successful at simulating the pathogen's *in vivo *metabolic state. In order to investigate metabolic requirements for *in vivo *growth, the web-based version of the *M. tuberculosis *GSMN is able to optimize for two different objective functions: the first specifying a minimal biomass with components essential for *in vitro *growth, and the second specifying a complete biomass composition that includes synthesis of virulence factors such as sulfolipid and dimycocerosate esters. Even if the growth rate of the pathogen does not reach its maximal value, simulation using the complete biomass composition in the GSMN-TB will still produce valuable qualitative predictions concerning essentiality of genes and reactions required for *in vivo *growth. These qualitative results will be useful in predicting drug targets, even in the situation in which the actual growth rate of the pathogen is lower than that predicted by the model.

Our web-based software makes, for the first time, genome-scale metabolic simulations available to the nonspecialist. It is therefore a valuable resource for biologists investigating the physiology and pathogenicity of *M. tuberculosis*. This resource will be developed by continuous curation of the metabolic model, leading to improved gene annotation, incorporation of high throughput datasets, and direct experimental testing of hypotheses generated by the model.

## Materials and methods

### Bacterial strains and growth conditions

*M. bovis *BCG strain (ATCC 35748) was cultured in a 2-l bioreactor (Adaptive Biosystem Voyager, Adaptive Biosystems Ltd, Luton, UK) under aerobic conditions and at pH 6.6, as previously described [25]. Chemostat cultures were grown in Roisin's minimal medium at a constant dilution rate of 0.02 per hour (equivalent to a doubling time of 34.7 hours). Steady-state conditions were assumed when the carbon dioxide evolution, optical density at 600 nm, and DW remained constant for three consecutive volume changes. Once the steady state was reached, cells were harvested for analysis. Biomass was determined according to the method described by Lynch and Bushell [48]. The amounts of glycerol in the supernatant and in fresh medium were assayed by use of a commercial assay kit that employs a glycerokinase-coupled enzyme assay system (Boehringer Mannheim, Mannheim, Germany).

### Assay of isocitrate lyase activity

Crude enzyme extracts were prepared as described by Honer zu Bentrup and coworkers [49] with minor modifications. Cells were harvested, washed three times with ice cold phosphate-buffered saline and resuspended in MOPS buffer (50 mmol/l MOPS (morphilinepropane sulfonate) [pH 6.8], 5 mmol/l MgCl_2_, 5 mmol/l L-cysteine, 1 mmol/l EDTA) supplemented with protease inhibitors (Complete™; Roche, Welwyn Garden City, UK). The cells were disrupted using a Ribolyser (Hybaid, Hybaid Ltd., Ashford, Kent, UK); speed setting 6.5 for 30 s) with careful cooling between each cycle. A lactose dehydrogenase coupled continuous method was used to assay isocitrate lyase activity [50].

### Construction of the GSMN-TB

The genome-scale model of the related actinomycete *Streptomyces coelicolor *[21] was used as the starting point for the construction of the genome-scale metabolic network (GSMN) of *Mycobacterium tuberculosis*. The gene orthology clusters computed by the KEGG database [51] were used to assign the respective *M. tuberculosis *H37Rv gene numbers to the genes present in the *S. coelicolor *model. The KEGG and MtbRvCyc (part of BioCyc) databases were used to incorporate additional *M. tuberculosis *specific reactions that do not have *S. coelicolor *counterparts. In situations in which a reaction was essential to produce a viable *in silico *model but the gene was not annotated in the genome, the reaction formulae was included in the model without genomic evidence. A significant proportion of the model was manually generated from journal publications describing dedicated experimental work (Table 1). For instance, the route for glycerol utilization is generally assumed to proceed via glycerol kinase followed by dehydrogenation [26]. However, many bacteria utilize an alternative pathway whereby glycerol is first oxidized by glycerol dehydrogenase before being phosphorylated [52]. Glycerol dehydrogenase activity has been detected in *M. tuberculosis *[53,54], but no gene encoding this activity has been annotated in the genome. Several genes encoding putative alcohol dehydrogenases are present, which could oxidize glycerol to glyceraldehyde (EC 1.1.1.72) to be further oxidized and then phosphorylated before entering glycolysis. This pathway is also included in the model.

### Flux balance analysis

Computer simulation protocols that are used in this work and made available in our interactive web resource are based on FBA. The principles of FBA are described in detail elsewhere [16,55]; here we briefly present the basic assumptions for the sake of introducing notation. The stoichiometric model of GSMN-TB was represented by the following equation:

b = S × v

Where b = (dc_1_/dt, ..., dc_m_/dt) is the vector of concentration changes of m metabolites (c_i _being the concentration of the i^th ^metabolite); v is the vector of metabolic fluxes carried out by *n *reactions in the network; and S is the *m *× *n *matrix of the stoichiometric coefficients. Fluxes are further subjected to the capacity constraints: α_j _≤ v_j _≤ β_j _(where α_j _and β_j _are the lower and upper bounds of the flux carried by the j^th ^reaction). The bounds of irreversible reactions were set to α_j _= 0 and β_j _= +∞; the bounds of reversible reactions were set to α_j _= -∞ and β_j _= +∞, allowing these reactions to carry either positive or negative flux. Following FBA methodology, we have studied metabolism under steady-state conditions in which there is no accumulation of intracellular (internal) metabolites, and concentrations of these metabolites do not change:

bi=0=∑j=1mSijvj∧cj∈I
 MathType@MTEF@5@5@+=feaafiart1ev1aaatCvAUfeBSjuyZL2yd9gzLbvyNv2Caerbhv2BYDwAHbqedmvETj2BSbqee0evGueE0jxyaibaiKI8=vI8tuQ8FMI8Gi=hEeeu0xXdbba9frFj0=OqFfea0dXdd9vqai=hGuQ8kuc9pgc9s8qqaq=dirpe0xb9q8qiLsFr0=vr0=vr0dc8meaabaqaciGacaGaaeqabaqadeqadaaakeaacaqGIbWaaSbaaSqaaiaabMgaaeqaaOGaeyypa0JaaGimaiabg2da9maaqahabaGaae4uamaaBaaaleaacaqGPbGaaeOAaaqabaGccaqG2bWaaSbaaSqaaiaabQgaaeqaaaqaaiaabQgacqGH9aqpcaaIXaaabaGaaeyBaaqdcqGHris5aOGaey4jIKTaae4yamaaBaaaleaacaqGQbaabeaakiabgIGiolaabMeaaaa@48D7@

Where I denotes the set of internal metabolites. Extracellular (external) metabolites transported from or secreted to the environment were not required to obey the balance equation (Equation 2) and were considered to have unlimited sources and sinks. Transport reactions for nutrient sources, which were rate limiting in chemostat experiments, were constrained to the experimentally determined flux values. In addition, a pseudo-reaction has been added to the system to model growth (biomass synthesis). The growth reaction has only one product, representing biomass, where the substrates correspond to biomass components and real-valued stoichiometric coefficients represent biomass composition. The flux through this reaction is equal to the growth rate of the bulk cell culture.

Linear programming was used to determine maximal theoretical flux toward a selected (internal or external) metabolite Z in the network, consistent with constraints imposed by stoichiometric matrix, balance conditions, and capacity bounds. The following linear programming problem was solved.

Maximize Z such that:

Z=bz=∑j=1nSzjvjb=S×v∀i∈I, bi=0, i≠zαi≤vi≤βi
 MathType@MTEF@5@5@+=feaafiart1ev1aaatCvAUfeBSjuyZL2yd9gzLbvyNv2Caerbhv2BYDwAHbqedmvETj2BSbqee0evGueE0jxyaibaiKI8=vI8tuQ8FMI8Gi=hEeeu0xXdbba9frFj0=OqFfea0dXdd9vqai=hGuQ8kuc9pgc9s8qqaq=dirpe0xb9q8qiLsFr0=vr0=vr0dc8meaabaqaciGacaGaaeqabaqadeqadaaakeaafaqaaeabbaaaaeaacaqGAbGaeyypa0JaaeOyamaaBaaaleaacaqG6baabeaakiabg2da9maaqahabaGaae4uamaaBaaaleaacaqG6bGaaeOAaaqabaGccaqG2bWaaSbaaSqaaiaabQgaaeqaaaqaaiaabQgacqGH9aqpcaaIXaaabaGaaeOBaaqdcqGHris5aaGcbaGaaeOyaiabg2da9iaabofacqGHxdaTcaqG2baabaGaeyiaIiIaaeyAaiabgIGiolaabMeacaGGSaGaaeiiaiaabkgadaWgaaWcbaGaaeyAaaqabaGccqGH9aqpcaaIWaGaaiilaiaabccacaqGPbGaeyiyIKRaaeOEaaqaaiaabg7adaWgaaWcbaGaaeyAaaqabaGccqGHKjYOcaqG2bWaaSbaaSqaaiaabMgaaeqaaOGaeyizImQaaeOSdmaaBaaaleaacaqGPbaabeaaaaaaaa@614E@

If the coefficients of optimization function Z have been set to the row of the stoichiometric matrix corresponding to biomass metabolite, the result of linear programming optimization represented the maximal theoretical growth rate.

The optimal value of the objective function calculated by FBA is unique, but associated metabolic flux distribution is not. There may be many flux distributions resulting in the same, optimal value of objective function. Therefore, flux distributions computed by single FBA simulation cannot be used to study internal metabolic state of the cell. The FVA method finds the full numerical range of each flux in all alternate flux distributions resulting in the same optimal value of objective function. During FVA simulation the objective function is constrained to its optimal value. Subsequently, the flux through each reaction in the model is subjected to minimization and maximization, resulting in minimal and maximal value defining the flux range. These values are unique and can be used to investigate metabolic state of the cell.

### Definition of growth requirements in vitro and in vivo

Biomass composition was estimated from reported data from a variety of sources (Additional data file 1). To account for the different growth requirements of *M. tuberculosis *growth *in vitro *and *in vivo*, we defined two biomass synthesis formulae with different sets of components required for growth. BIOMASS1 reflects the actual macromolecular composition of *M. tuberculosis*. BIOMASSe is a minimal macromolecular composition of *M. tuberculosis *and includes only those components thought to be essential for growth *in vitro*.

### Modeling of co-factor requirement of enzymatic reactions

Numerous enzymes require nonpeptide co-factors that are regenerated either within the reaction or in a coupled reaction. Co-factor utilization can provide useful clues to metabolic activity, and co-factor synthesis pathways may provide potential drug targets. Stoichiometric models of metabolism published thus far either do not include co-factors in reaction formulae or balance co-factor consumption reactions with reactions that regenerate the cofactor (as for NAD/NADH in our model). However, in FBA analysis this strategy has the effect of eliminating the need for co-factor synthesis. Although enzyme co-factors are not consumed by reactions, they have finite chemical stability and must be replenished by *de novo *synthesis. To force a flux toward synthesis, FBA models often include the co-factor in biomass composition but this has the effect of making synthesis of the co-factor constitutive in all conditions, which may be appropriate for essential co-factors such as NAD but is less so for co-factors such as molybdenum, which may be required only under certain conditions. To simulate the nonconstitutive requirement for these co-factors, we introduce the concept of a replenishing flux in which the nonconstitutive cofactors are included in reactions but with an arbitrary very low (0.001) stoichiometric coefficient toward consumption. Reversible reactions are written twice with co-factor consumption in both directions. This has the effect of forcing co-factor synthesis only in conditions where the co-factor utilizing reaction is active. The small replenishing flux toward co-factor synthesis has little influence on the magnitude of flux carried by the reaction and on the energy and mass balance of the metabolism, but it makes co-factor synthesis essential for the reaction to proceed.

### Model calibration by comparison with chemostat data

Calibration of the model involved determination of three energetic parameters. The first of these is the ratio of the number of ATP molecules formed to the number of O atoms reduced by electron transport (P/O ratio). The P/O ratio is set by the stoichiometric coefficients of the reactions involved in electron transport and ATP synthesis. The second is the growth-dissociated cost of polymerization of the building blocks into biologic polymers (DNA replication, transcription, translation, and so on; m_ATP_). To account for growth-dissociated cost, ATP dissipating reaction was included in the system and its flux was constrained to Y_xATP_. The final parameter is the cost of growth-associated maintenance (Y_xATP_), modeled by including ATP hydrolysis as a part of the biomass synthesis formula.

To compare quantitative model predictions with our chemostat data, we constrained the growth rate to a chemostat dilution rate and computed the minimal glycerol uptake rate. The stoichiometry of the electron transport chain was considered to be similar to that of *Corynebacterium glutamicum *[56]. The ATP yield coefficient Y_xATP _and the maintenance flux m_ATP _were systematically changed to achieve good agreement with chemostat data, following previously reported approaches [21].

### Gene essentiality prediction and comparison with TRASH data

For each gene we pre-computed the list of all reactions in the model that require the product of this gene, according to Boolean rules describing gene-protein relationships. Subsequently, the effect of gene inactivation on growth was predicted by constraining the fluxes through all reactions requiring this gene to 0 and running linear programming to determine the resulting maximal theoretical growth rate. If the resulting growth rate was less than 0.001, then the gene was considered to be essential for growth.

Computational predictions were compared with the findings of TraSH mutagenesis experiments [28,57]. Transport reactions were constrained to reproduce the experimental conditions utlilized in the TraSH mutagenesis experiment (7H10 agar composition) and essential genes were computed. The list of genes that were predicted to be essential *in vitro *was compared with the list of *in vitro *essential genes identified by TraSH mutagenesis [28]. To test whether model predictions are closer to the TraSH mutagenesis data than expected by chance, we applied a Fisher exact test. The null hypothesis of this test, as applied to our data, states that gene essentiality assignments performed by the GSMN-TB model and TRASH experiments are independent.

### Implementation of calculations presented in this work and web-based software

Linear programming calculations and evaluation of Boolean expressions representing gene-protein associations were implemented in C programming language using GNU Linear Programming Kit library [58]. The web interface was implemented as a collection of Perl-CGI scripts running computational module under Linux operating system. Data handling during model building was performed in MS Excel, Perl, Python, and MySQL. Comparison of gene essentiality predictions with TRASH data was implemented in R [59].

## Additional data files

The following additional data are available with the online version of this paper. Additional data file 1 illustrates the estimated macromolecular composition for *M. tuberculosis*. Additional data file 2 shows the calculations used to estimate that composition. Additional data file 3 shows the conversion between stoichiometric formulae and mmol/l per gram of biomass. Additional data file 4 shows reaction formulae, limits, Enzyme Commission (EC) numbers, genes, and pathway classifications. Additional data file 5 provides references for those reactions. Additional data file 6 provides metabolite names. Additional data file 7 contains instructions on how to use the GSMN-TB server, illustrated by screenshots.

## Supplementary Material

Additional data file 1 Illustrated is the estimated macromolecular composition for *M. tuberculosis*. Click here for file

Additional data file 2 Shown are the calculations used to estimate the composition shown in Additional data file 1. Click here for file

Additional data file 3 Shown is the conversion between stoichiometric formulae and mmol/l per gram of biomass. Click here for file

Additional data file 4 Shown are the reaction formulae, limits, EC numbers, genes, and pathway classifications. Click here for file

Additional data file 5 Provided are references for the reactions shown in Additional data file 4. Click here for file

Additional data file 6 Provided are metabolite names. Click here for file

Additional data file 7 Provided are instructions on how to use the GSMN-TB server, illustrated by screenshots. Click here for file
