# Supplementary material for: GSMN-TB: a web-based genome-scale network model of Mycobacterium tuberculosis metabolism
Source: Genome Biol. 2007 May 23;8(5):R89. doi: 10.1186/gb-2007-8-5-r89 (PMC1929162; doi:10.1186/gb-2007-8-5-r89)
Supplement: Additional data file 7 — Provided are instructions on how to use the GSMN-TB server, illustrated by screenshots. [file gb-2007-8-5-r89-S7.pdf]

Description of WWW interface to:

GSMN-TB: a web-based genome scale network model of *Mycobacterium tuberculosis* metabolism

Dany JV Beste[1]*, Tracy Hooper[1]*, Graham Stewart[1], Bhushan Bonde[1], Claudio Avignone-Rossa[1], Michael Bushell[1], Paul Wheeler[2], Steffen Klamt[3], Andrzej M. Kierzek[1]* and Johnjoe McFadden[1]*.

1. Front page.

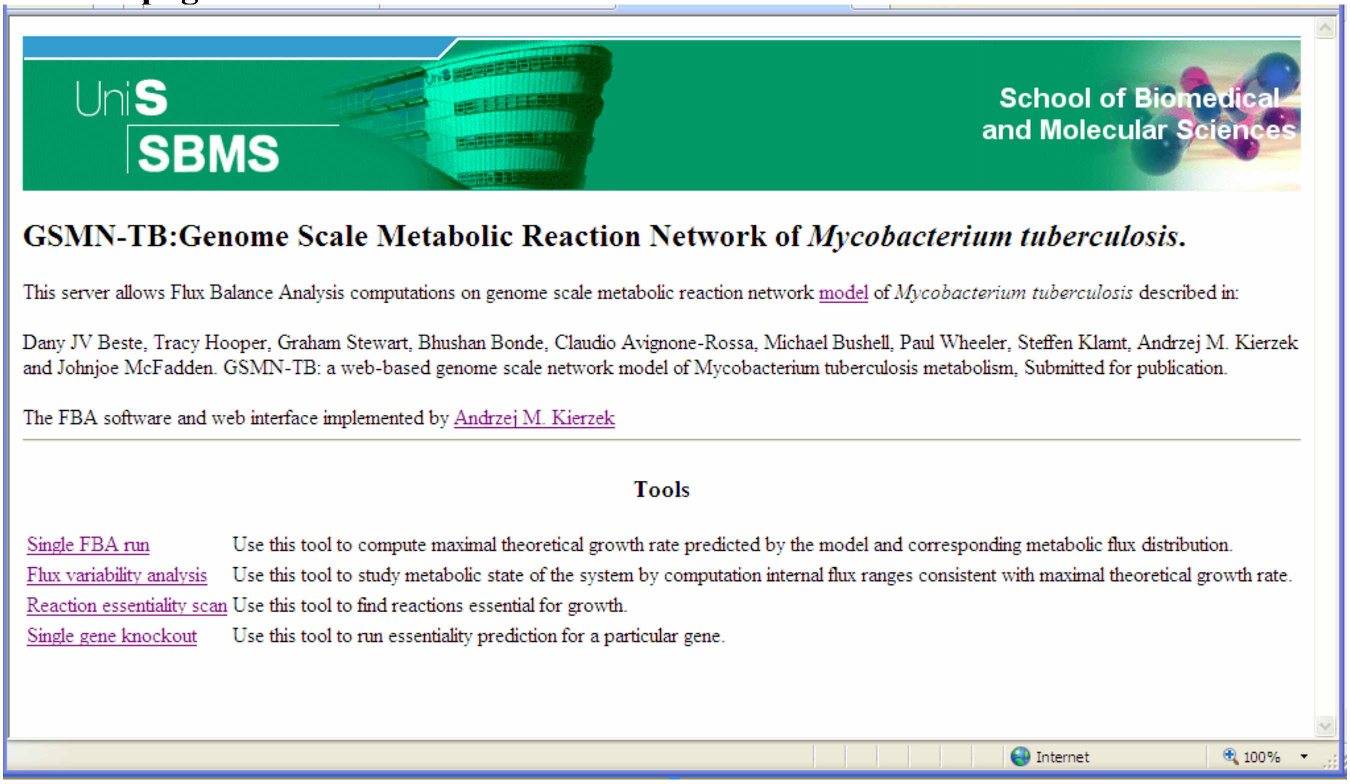

The front page of the interface contains links to four constraint-based simulation methods implemented in our software. Chose one of the following:

Method	Application
Single FBA run	Computes maximal theoretical growth rate and one of many metabolic flux distributions sustaining the growth with this rate.
Flux Variability Analysis	For each reaction the software computes the range of metabolic flux in all alternative flux distributions that sustain the growth with maximal theoretical rate.
Reaction Essentiality Scan	Each reaction in turn is removed from the system and the maximal theoretical growth rate is computed. Reactions for which the maximal growth rate is 0 or the system is not declared to be OPTIMAL are predicted to be essential.
Single gene knockout	Predicts essentiality for selected gene present in the model.

2. Model page.

Genome scale metabolic reaction network of *Mycobacterium tuberculosis*.

See this file for full names of metabolites occurring in reaction formulas.

Reaction name	Reaction formula	Lower bound	Upper bound	Genes
R1	1 GL + 1 ATP = 1 ADP + 1 GL3P	0	100000	Rv3696c
R2	1 GL + 1 NADP = 1 T3 + 1 NADPH	-100000	100000	Rv2982cOR Rv0564OR Rv3045
R3	1 GL + 1 NAD = 1 T3 + 1 NADH	-100000	100000	Rv0162c
R4	1 GL3P + 1 MK = 1 DHAP + 1 MKH2	0	100000	Rv3302cOR Rv2249c
R5	1 T3 + 1 NAD = 1 DGLYCERATE + 1 NADH	0	100000	Rv2858cOR Rv0458
R6	1 DGLYCERATE + 1 ATP = 1 3PG + 1 ADP	0	100000	Rv2205c
R7	1 RIB + 1 ATP = 1 ADP + 1 R5P	0	100000	Rv2436
R8	1 F1P = 1 DHAP + 1 T3	0	100000	Rv0363c
R9	1 FRU = 1 GLC	-100000	100000	orphan
R10	1 FRU = 1 MAN	-100000	100000	orphan
R11	1 MAN6P = 1 F6P	-100000	100000	Rv3255c
R12	1 MAN1P = 1 MAN6P	-100000	100000	Rv3257cOR Rv3308
R13	1 MAN1P + 1 GTP = 1 GDPMAN + 1 PI	0	100000	Rv3264c
R14	1 GDPMAN + 1 DPP = 1 GDP + 1 PPM	0	100000	Rv2051c
R15	1 GDPMAN = 1 GDPDHDOMAN	0	100000	Rv0112OR Rv1511
R16	1 GDPDHDOMAN = 1 GDPDHDOGAL	0	100000	Rv1512

Internet 100%

Both front page of the system and the pages of individual simulation methods contain a link to the model page displaying the genome scale metabolic reaction network model of *M. tuberculosis*. The first column of the table lists the reaction name which is used in the definition of constraints for simulation methods. The second column lists reaction formula. The link to the file with full names of metabolites used in formulas can be found at the top of the page. Columns three and four list default capacity constraints of individual reactions. Finally, the last column of the file lists gene annotation. The annotation has the form of a Boolean statement. The OR operator indicates that genes (or groups of genes) encode isoenzymes catalysing the same reaction. The AND statement indicates that genes (or groups of genes) encode subunits of the same enzyme. The Boolean statements are used to determine which reactions should be inactivated in gene essentiality simulations. The genes are named by Rv numbers linked to appropriate gene pages in the TubercuList database. The reactions for which genes are unknown are annotated as “orphan”.

3. Simulation setup pages.

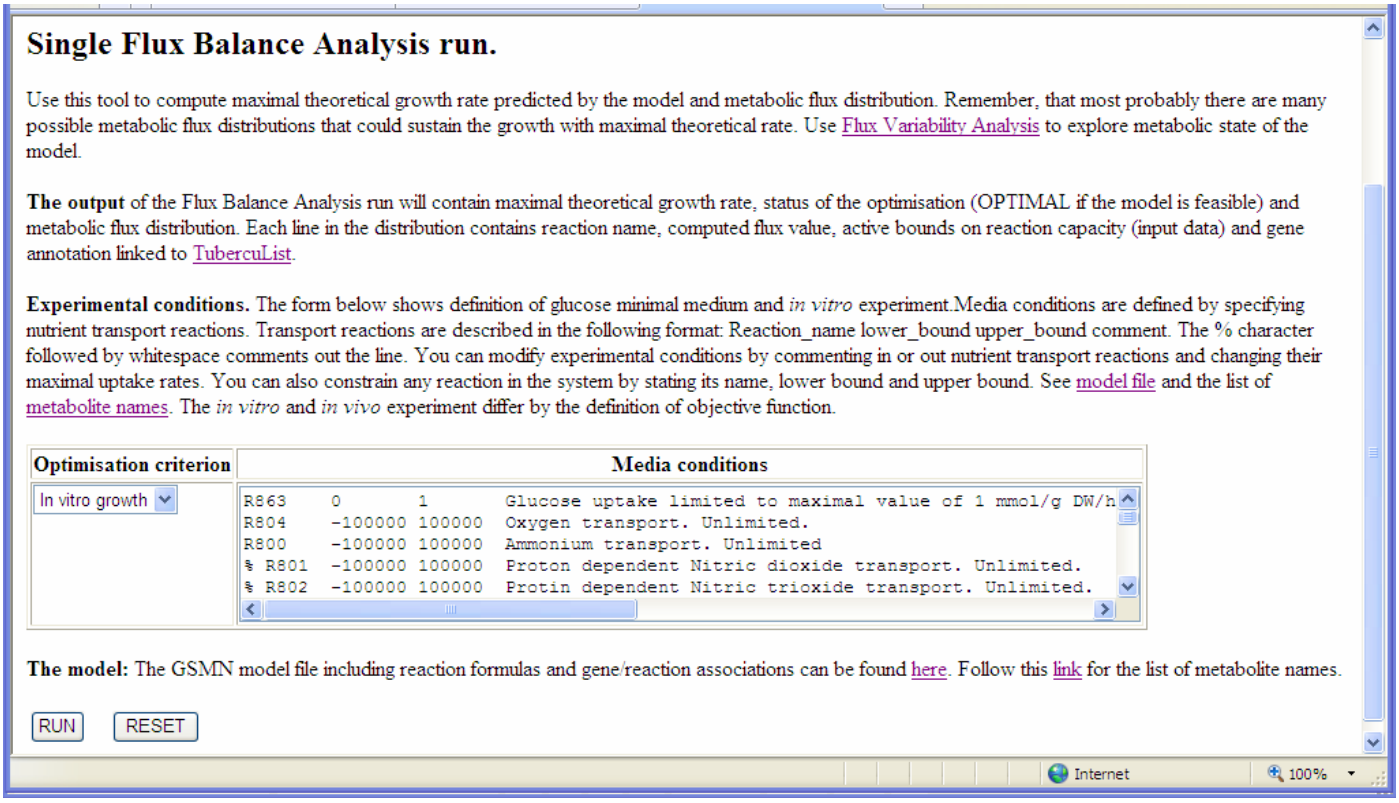

Single Flux Balance Analysis run.

Use this tool to compute maximal theoretical growth rate predicted by the model and metabolic flux distribution. Remember, that most probably there are many possible metabolic flux distributions that could sustain the growth with maximal theoretical rate. Use Flux Variability Analysis to explore metabolic state of the model.

The output of the Flux Balance Analysis run will contain maximal theoretical growth rate, status of the optimisation (OPTIMAL if the model is feasible) and metabolic flux distribution. Each line in the distribution contains reaction name, computed flux value, active bounds on reaction capacity (input data) and gene annotation linked to TubercuList.

Experimental conditions. The form below shows definition of glucose minimal medium and *in vitro* experiment.Media conditions are defined by specifying nutrient transport reactions. Transport reactions are described in the following format: Reaction_name lower_bound upper_bound comment. The % character followed by whitespace comments out the line. You can modify experimental conditions by commenting in or out nutrient transport reactions and changing their maximal uptake rates. You can also constrain any reaction in the system by stating its name, lower bound and upper bound. See model file and the list of metabolite names. The *in vitro* and *in vivo* experiment differ by the definition of objective function.

Optimisation criterion	Media conditions
In vitro growth	R863 0 1 Glucose uptake limited to maximal value of 1 mmol/g DW/h<br>R804 -100000 100000 Oxygen transport. Unlimited.<br>R800 -100000 100000 Ammonium transport. Unlimited<br>% R801 -100000 100000 Proton dependent Nitric dioxide transport. Unlimited.<br>% R802 -100000 100000 Protin dependent Nitric trioxide transport. Unlimited.

The model: The GSMN model file including reaction formulas and gene/reaction associations can be found here. Follow this link for the list of metabolite names.

RUN RESET

Internet 100%

The figure above shows the simulation set up page which appears after clicking “Single FBA run” link on the front page of our system. The page contains a short description of the simulation method (Single Flux Balance Analysis run in this case), its output and description of simulation setup. The form below allows the choice of optimisation criterion reflecting *in vitro* or *in vivo* growth requirements and media conditions. The simulation setup pages for Single FBA simulation, Flux Variability Analysis and Reaction Essentiality Scan are identical. The simulation setup page for simulation of single gene knockout additionally allows selection of the gene to be inactivated.

Optimisation criterion: The optimisation criteria for *in vivo* and *in vitro* growth differ in the definition of biomass synthesis reaction which is used as objective function in linear programming calculations. To account for the different growth requirements of *M. tuberculosis* growth *in vitro* and *in vivo* we defined two biomass synthesis formulae with different sets of components required for growth. BIOMASS1 reflects the actual macromolecular composition of *M. tuberculosis*. BIOMASSe is a minimal macromolecular composition of *M. tuberculosis* and includes only those components thought to be essential for growth *in vitro*.

Definition of media conditions and other constraints.

The “Media conditions” form allows the user to change default capacity constraints of **any reaction in the model**. The constraint is set by simply typing in the line containing the reaction name, lower bound and upper bound separated by whitespace characters. The following part of the line is treated as a comment.

The most frequent application of this form is the set up of media conditions by changing capacity constraints of transport reactions. All transport fluxes are set to 0 in the default version of the model. Thus to run a simulation the user must overload this settings by allowing the transport of selected nutrient sources. The form already contains the minimal glucose medium which serves as an example of input format. It also contains commented out lines for other transport reactions in the model. If the first character of the line is % followed by whitespace character the whole line is treated as a comment. The open a particular gate the user should delete the % followed by whitespace.

To constraint reactions other than one of transport reactions listed in the form you need to find its name in the model file. The link to model file is available below the form.

Example: Changing carbon source from glucose to glycerol.

1. Comment out the line corresponding to glucose transport to constraint the flux of glucose transport to the default value of 0 (alternatively, change the upper bound from 1 to 0).

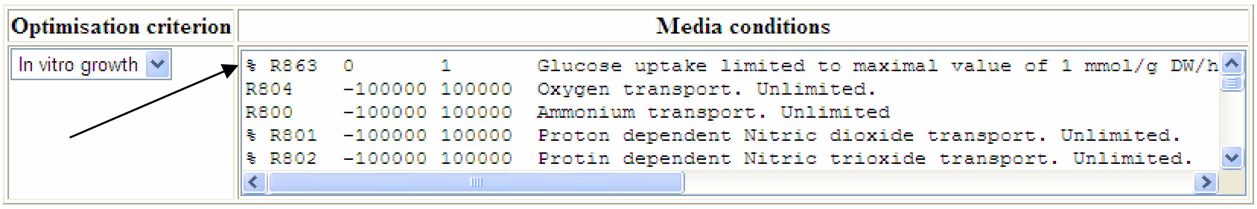

2. Find the line corresponding to glycerol transport, remove the comment character and set upper bound:

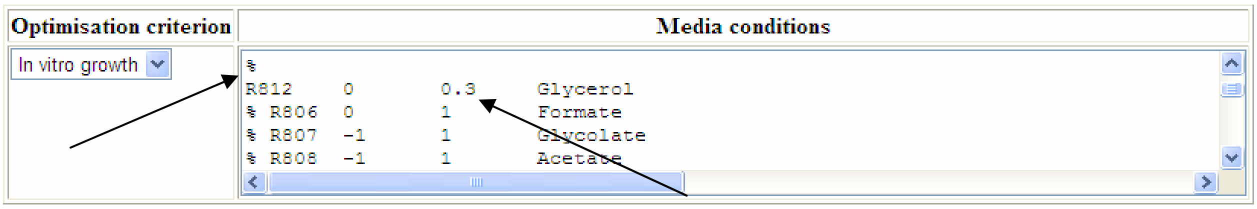

The example lines for carbon sources contain the upper bound value set to 1 mmol/g DW/h. In this example we changed this value to a glycerol transport rate of 0.3 mmol/g DW/h.

The RUN button starts the simulation.

4. The single FBA run.

Optimisation status: OPTIMAL

Maximal theoretical growth rate: 0.048522

One of possibly many flux distributions sustaining maximal theoretical growth rate:

Reaction name	Flux	Reaction formula	Lower bound	Upper bound	Genes
R1	0.000000	1 GL + 1 ATP = 1 ADP + 1 GL3P	0	100000	Rv3696c
R2	-100000.000000	1 GL + 1 NADP = 1 T3 + 1 NADPH	-100000	100000	Rv2982cOR Rv0564OR Rv3045
R3	100000.000000	1 GL + 1 NAD = 1 T3 + 1 NADH	-100000	100000	Rv0162c
R4	0.000000	1 GL3P + 1 MK = 1 DHAP + 1 MKH2	0	100000	Rv3302cOR Rv2249c
R5	0.000000	1 T3 + 1 NAD = 1 DGLYCERATE + 1 NADH	0	100000	Rv2858cOR Rv0458
R6	0.000000	1 DGLYCERATE + 1 ATP = 1 3PG + 1 ADP	0	100000	Rv2205c
R7	0.000000	1 RIB + 1 ATP = 1 ADP + 1 R5P	0	100000	Rv2436
R8	0.000000	1 F1P = 1 DHAP + 1 T3	0	100000	Rv0363c
R9	0.000000	1 FRU = 1 GLC	-100000	100000	orphan
R10	0.000000	1 FRU = 1 MAN	-100000	100000	orphan
R11	-0.005653	1 MAN6P = 1 F6P	-100000	100000	Rv3255c
R12	-0.005653	1 MAN1P = 1 MAN6P	-100000	100000	Rv3257cOR Rv3308
[illegible]	[illegible]	[illegible]	[illegible]	[illegible]	[illegible]

The example “Single FBA run” simulation produces the output page shown above. The top of the file lists the maximal theoretical growth rate and the status of Linear Programming optimisation. If the status is different than OPTIMAL then the system is considered to be infeasible. This means that some balance equations or capacity constraints are violated and the only valid conclusion about the system is that it cannot reproduce growth. In this situation the numerical value of the maximal theoretical growth rate does not have any biological significance.

The table on the output page shows one of many possible metabolic flux distributions that sustains the maximal theoretical growth rate. Each row in the table contains reaction name, the value of metabolic flux through this reaction, reaction formula, the capacity constraints (lower and upper bound) active during simulation and the gene annotation linked to TubercuList.

The “Single FBA run” simulation is fast. The time for appearance of the output page is limited only by the speed of the network connection.

5. Flux Variability Analysis

Maximal theoretical growth rate: 0.04852171175

Flux ranges consistent with maximal theoretical growth rate:

	Minimal flux	Maximal flux	Reaction formula	Genes
R1	0	0.001340096247	1 GL + 1 ATP = 1 ADP + 1 GL3P	Rv3696c
R2	-100000	100000	1 GL + 1 NADP = 1 T3 + 1 NADPH	Rv2982cOR Rv0564OR Rv304[illegible]
R3	-100000	100000	1 GL + 1 NAD = 1 T3 + 1 NADH	Rv0162c
R4	0	0.05025732928	1 GL3P + 1 MK = 1 DHAP + 1 MKH2	Rv3302cOR Rv2249c
R5	0	0.001305291061	1 T3 + 1 NAD = 1 DGLYCERATE + 1 NADH	Rv2858cOR Rv0458
R6	0	0.001305291061	1 DGLYCERATE + 1 ATP = 1 3PG + 1 ADP	Rv2205c
R7	0	0.05025732928	1 RIB + 1 ATP = 1 ADP + 1 R5P	Rv2436
R8	0	0	1 F1P = 1 DHAP + 1 T3	Rv0363c
R9	0	0	1 FRU = 1 GLC	orphan
R10	0	0	1 FRU = 1 MAN	orphan
R11	-0.00565258537	-0.005646932741	1 MAN6P = 1 F6P	Rv3255c
R12	-0.00565258537	-0.005646932742	1 MAN1P = 1 MAN6P	Rv3257cOR Rv3308
R13	0.005646932742	0.00565258537	1 MAN1P + 1 GTP = 1 GDPMAN + 1 PI	Rv3264c
R14	0.003508218042	0.003511729799	1 GDPMAN + 1 DPP = 1 GDP + 1 PPM	Rv2051c

Waiting for http://sysbio.sbs.surrey.ac.uk/cgi-bin/tbfluxvar1... Internet 100%

The example "Flux Variability Analysis" simulation results in the page shown above. The top of the page shows maximal theoretical growth rate. Each row in the page below corresponds to one reaction and shows minimal and maximal flux values for this reaction if the growth rate is constrained to the maximal theoretical value printed at the top of the page. The fields in the row are: reaction name, minimal flux of reaction, maximal flux of reaction, reaction formula, gene annotation linked to TubercuList.

Contrary to the flux values computed in single FBA run the FVA flux ranges are unique and may be used to draw conclusion about the metabolic state of the cell.

The FVA calculations are relatively slow as two Linear Programming optimisations of the entire model are run for each reaction. The rows of the table are displayed in the browser as calculation progresses and the simulation is usually finished within 10 minutes.

6. Reaction Essentiality Scan

Each row in the table represents FBA simulation in which reaction has been removed from the system. Thus reactions for which the maximal growth rate is 0 or the optimisation status is different from OPTIMAL are predicted to be essential for growth.

<

	Optimisation status	Maximal growth rate	Reaction formula removed from the system	Genes
R1	OPTIMAL	0.04852171208	1 GL + 1 ATP = 1 ADP + 1 GL3P	Rv3696c
R2	OPTIMAL	0.04852171208	1 GL + 1 NADP = 1 T3 + 1 NADPH	Rv2982cOR Rv0564OR Rv304[illegible]
R3	OPTIMAL	0.04852171216	1 GL + 1 NAD = 1 T3 + 1 NADH	Rv0162c
R4	OPTIMAL	0.04852171208	1 GL3P + 1 MK = 1 DHAP + 1 MKH2	Rv3302cOR Rv2249c
R5	OPTIMAL	0.04852178164	1 T3 + 1 NAD = 1 DGLYCERATE + 1 NADH	Rv2858cOR Rv0458
R6	OPTIMAL	0.04852178164	1 DGLYCERATE + 1 ATP = 1 3PG + 1 ADP	Rv2205c
R7	OPTIMAL	0.04852171289	1 RIB + 1 ATP = 1 ADP + 1 R5P	Rv2436
R8	OPTIMAL	0.04852171175	1 F1P = 1 DHAP + 1 T3	Rv0363c
R9	OPTIMAL	0.04852171175	1 FRU = 1 GLC	orphan
R10	OPTIMAL	0.04852171175	1 FRU = 1 MAN	orphan
R11	OPTIMAL	8.583985716e-12	1 MAN6P = 1 F6P	Rv3255c
R12	OPTIMAL	0	1 MAN1P = 1 MAN6P	Rv3257cOR Rv3308
R13	OPTIMAL	0	1 MAN1P + 1 GTP = 1 GDPMAN + 1 PI	Rv3264c

Waiting for http://sysbio.sbs.surrey.ac.uk/cgi-bin/tbwebrscan1... Internet 100%

Example reaction essentiality scan generates the page shown above. Each field in the table corresponds to a reaction. During simulation the flux through the reaction is constrained to 0 and the maximal theoretical growth rate is computed. The row of the table shows the reaction name, optimisation status, maximal theoretical growth rate, reaction formula and gene annotation linked to TubercuList. Reactions for which maximal theoretical growth rate is close to 0 or for which optimisation status is different than OPTIMAL are predicted to be essential for growth.

The Reaction Essentiality Scan is relatively slow as a Linear Programming optimisation of the entire model is run for each reaction. The rows of the table are displayed in the browser as the calculation progresses and the simulation is usually finished within 5 minutes.

7. Single gene knockout.

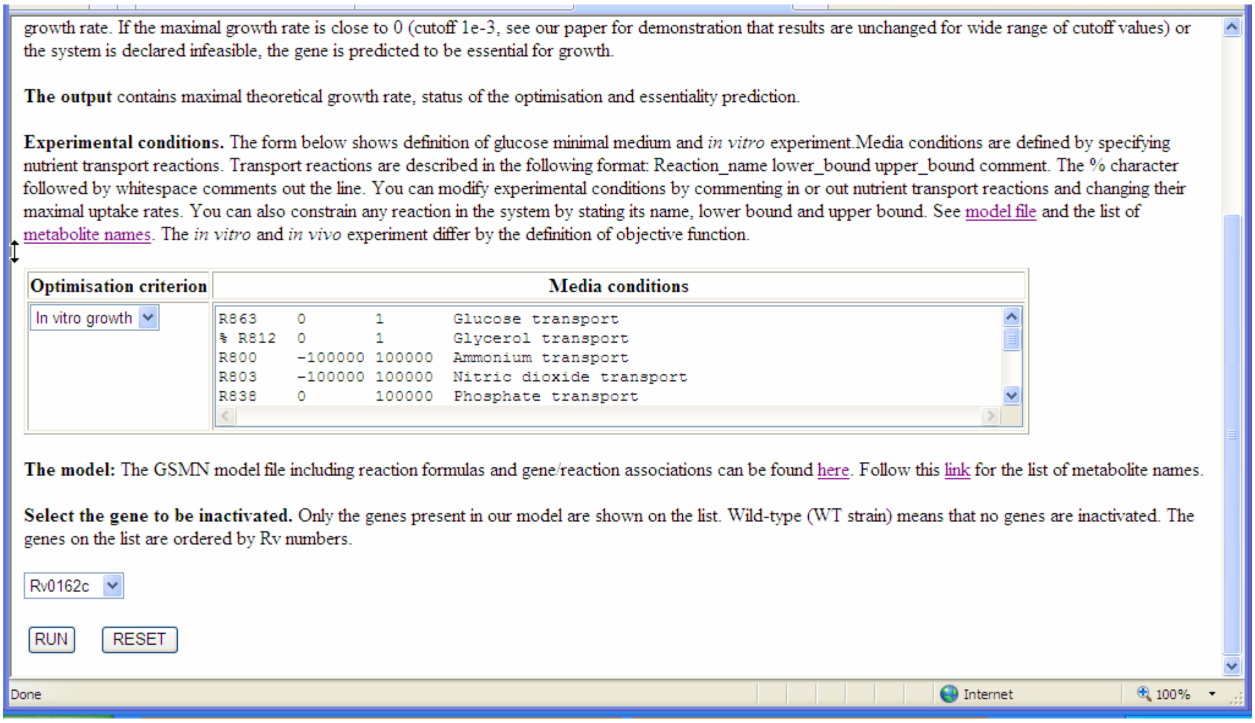
growth rate. If the maximal growth rate is close to 0 (cutoff 1e-3, see our paper for demonstration that results are unchanged for wide range of cutoff values) or the system is declared infeasible, the gene is predicted to be essential for growth.

The output contains maximal theoretical growth rate, status of the optimisation and essentiality prediction.

Experimental conditions. The form below shows definition of glucose minimal medium and *in vitro* experiment.Media conditions are defined by specifying nutrient transport reactions. Transport reactions are described in the following format: Reaction_name lower_bound upper_bound comment. The % character followed by whitespace comments out the line. You can modify experimental conditions by commenting in or out nutrient transport reactions and changing their maximal uptake rates. You can also constrain any reaction in the system by stating its name, lower bound and upper bound. See model file and the list of metabolite names. The *in vitro* and *in vivo* experiment differ by the definition of objective function.

Optimisation criterion	Media conditions
In vitro growth	R863 0 1 Glucose transport<br>% R812 0 1 Glycerol transport<br>R800 -100000 100000 Ammonium transport<br>R803 -100000 100000 Nitric dioxide transport<br>R838 0 100000 Phosphate transport

The model: The GSMN model file including reaction formulas and gene/reaction associations can be found here. Follow this link for the list of metabolite names.

Select the gene to be inactivated. Only the genes present in our model are shown on the list. Wild-type (WT strain) means that no genes are inactivated. The genes on the list are ordered by Rv numbers.

Rv0162c

RUN RESET

Done Internet 100%

The simulation setup page for Single Gene Knockout contains an additional selection box allowing specification of the gene to be inactivated. The selection lists contains all genes in the model ordered according to their Rv numbers. Wild type denotes the model with all genes active.

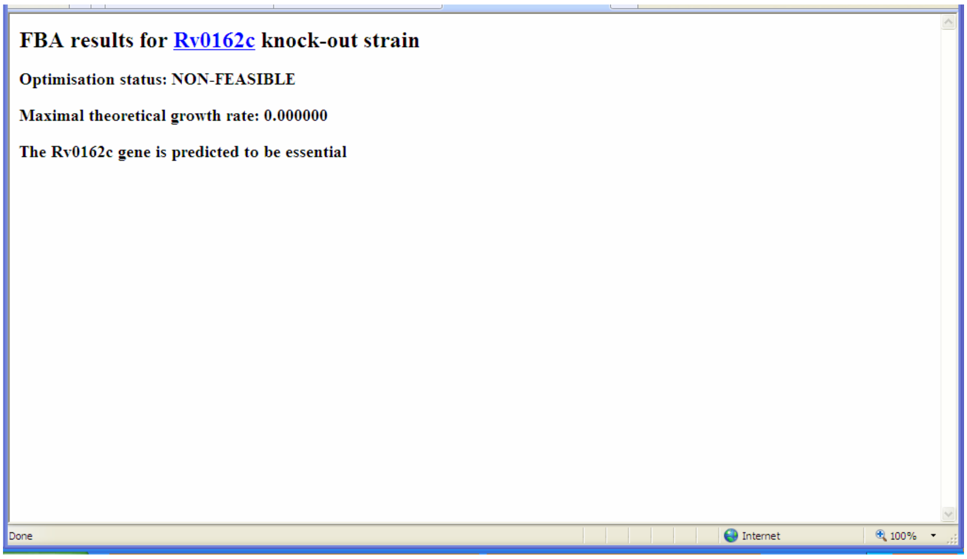
FBA results for Rv0162c knock-out strain

Optimisation status: NON-FEASIBLE

Maximal theoretical growth rate: 0.000000

The Rv0162c gene is predicted to be essential

Done Internet 100%

The page above shows an example of results. All reactions that require the product of the inactivated gene are constrained to 0 and the maximal theoretical growth rate is computed. The page displays gene name linked to TubercuList, optimisation status, maximal theoretical growth rate and the statement about gene essentiality. The genes for which growth rate is close to 0 or optimisation status is different from optimal are declared to be essential.
